# High Diagnostic Accuracy of Arterial Phase CT in Differentiating Pheochromocytoma in Good/Poor Washout Adrenal Masses

**DOI:** 10.1210/jendso/bvae199

**Published:** 2024-11-12

**Authors:** Aditya Phadte, Brijesh Krishnappa, Saba Samad Memon, Virendra Patil, Anurag Lila, Padma Vikram Badhe, Vijaya Sarathi, Gwendolyn Fernandes, Sameer Rege, Gagan Prakash, Santosh Menon, Manjiri Karlekar, Rohit Barnabas, Nalini Shah, Hemangini Thakkar, Tushar Bandgar

**Affiliations:** Department of Endocrinology, Seth G.S Medical College & KEM Hospital, Mumbai, Maharashtra 4000012, India; Department of Endocrinology, Seth G.S Medical College & KEM Hospital, Mumbai, Maharashtra 4000012, India; Department of Endocrinology, Seth G.S Medical College & KEM Hospital, Mumbai, Maharashtra 4000012, India; Department of Endocrinology, Seth G.S Medical College & KEM Hospital, Mumbai, Maharashtra 4000012, India; Department of Endocrinology, Seth G.S Medical College & KEM Hospital, Mumbai, Maharashtra 4000012, India; Department of Radiology, Seth G.S Medical College & KEM Hospital, Mumbai, Maharashtra 4000012, India; Department of Endocrinology, Vydehi Institute of Medical Sciences and Research Centre, Bangalore, Karnataka 560066, India; Department of Pathology, Seth G.S Medical College & KEM Hospital, Mumbai, Maharashtra 4000012, India; Department of General Surgery, Seth G.S Medical College & KEM Hospital, Mumbai, Maharashtra 4000012, India; Department of Uro-Oncology, Tata Memorial Hospital and Advanced Centre for Treatment Research and Education in Cancer, Homi Bhabha National Institute, Mumbai, Maharashtra 400012, India; Department of Pathology, Tata Memorial Hospital and Advanced Centre for Treatment Research and Education in Cancer, Homi Bhabha National Institute, Mumbai, Maharashtra 400012, India; Department of Endocrinology, Seth G.S Medical College & KEM Hospital, Mumbai, Maharashtra 4000012, India; Department of Endocrinology, Seth G.S Medical College & KEM Hospital, Mumbai, Maharashtra 4000012, India; Department of Endocrinology, Seth G.S Medical College & KEM Hospital, Mumbai, Maharashtra 4000012, India; Department of Radiology, Seth G.S Medical College & KEM Hospital, Mumbai, Maharashtra 4000012, India; Department of Endocrinology, Seth G.S Medical College & KEM Hospital, Mumbai, Maharashtra 4000012, India

**Keywords:** lipid poor adrenal mass, pheochromocytoma, arterial attenuation, bolus tracked, washout

## Abstract

**Introduction:**

Differentiating pheochromocytomas from other adrenal masses based on computed tomography (CT) characteristics remains challenging, particularly in lipid-poor lesions with variable washout patterns. This study evaluated CT features for distinguishing pheochromocytomas in good and poor washout subcohorts.

**Methods:**

We prospectively analyzed 72 patients with unilateral lipid-poor adrenal masses. CT protocol included basal, bolus-tracked arterial, early venous (45 seconds postarterial), and delayed (15 minutes postarterial) phases. Masses were categorized into good and poor washout groups. Histopathology provided the final diagnosis. CT characteristics and calculated indices were compared between pheochromocytomas and other masses in each washout category.

**Results:**

The cohort included pheochromocytomas (n = 35), adrenocortical carcinoma (ACC; n = 15), lipid-poor adenomas (n = 13), and metastatic infiltration/infection (n = 9). In the good washout group, pheochromocytomas (n = 15) showed larger diameters (6.00 vs 3.45 cm, *P* = .001), higher necrosis frequency (73.3% vs 30%, *P* = .049), and greater arterial attenuation (173.2 vs 74.5 HU, *P* < .001) compared to adenomas (n = 9). Arterial attenuation provided a high discriminatory value [area under the curve (AUC): 0.967], with 100% sensitivity at 87.6 Hounsfield unit (HU) and 100% specificity at 139.9 HU. In the poor washout group, pheochromocytomas (n = 20) exhibited higher arterial attenuation (99.2 vs 59.2 HU, *P* < .001; AUC: 0.844) compared to ACC (n = 14), metastatic infiltration (n = 9), and adenomas (n = 4), with 85% sensitivity and 78% specificity at 73.3 HU. Normetanephrine-secreting/nonsecretory pheochromocytomas showed higher arterial enhancement than metanephrine-secreting ones (132.0 vs 90.5 HU, *P* = .020) within the poor washout group.

**Conclusion:**

Arterial phase attenuation on CT has good diagnostic accuracy for differentiating pheochromocytomas from other adrenal masses in both good and poor washout categories, potentially guiding hormonal workup.

Computed tomography (CT) is a valuable tool for assessing adrenal masses, as various etiologies exhibit distinct CT characteristics. With the widespread use of CT for various indications, adrenal incidentalomas are becoming more common. Besides adrenal lesions like myelolipomas, cysts, and hemorrhages, which have specific CT features, a lipid-rich adrenal mass [<10 Hounsfield hnit (HU)] is highly suggestive of an adrenal adenoma and possesses a high specificity (98%) for differentiating it from nonadenoma masses [1, 2]. A recent study has proposed increasing the basal HU cutoff to 20 for differentiating adrenal masses [3].

For lipid-poor adrenal masses, good relative percentage washout (RPW) (≥40%) was considered specific for adrenal adenoma [4]. However, subsequent research revealed that among nonadenoma masses, a significant proportion of pheochromocytomas have good RPW [5, 6]. Hence, a lipid-poor adrenal mass with a good RPW can be mainly due to adenoma or pheochromocytoma. Larger datasets have shown that a substantial proportion of lipid-poor adenomas may have poor RPW (<40%) [7]. Therefore, a lipid-poor adrenal mass with poor RPW may be an adenoma, pheochromocytoma, adrenocortical carcinoma (ACC), or a metastasis/infiltrative lesion [8]. A recent study suggested higher cut-offs of 83% for absolute percentage washout and 58% for RPW to distinguish benign from malignant adrenal masses with very high specificity (>99%) but poor sensitivity (11-15%) [9].

Pheochromocytomas account for around 1% to 8% of adrenal incidentalomas on CT in endocrine setting [10]. Studies on CT characteristics have attempted to differentiate pheochromocytoma from adenoma with variable accuracies. An unenhanced HU value of less than 10 virtually excludes the possibility of pheochromocytoma [11]. However, such differentiation in subcohorts of lipid-poor adrenal masses with good washout and poor washout has not been attempted. Moreover, abdominal imaging for indications other than adrenal mass includes only baseline, arterial, and early venous phases. Hence, CT characteristics based on these phases to differentiate pheochromocytoma from other masses may offer cost-effective approaches for further evaluation. Therefore, we aimed to determine the utility of CT scan characteristics in suggesting the underlying etiology of adrenal masses (proven histologically) in different subcohorts based on washouts in a prospective manner.

## Materials and Methods

### Patients

The study was conducted at a tertiary healthcare institute in western India. The study protocol was approved by the Institutional Ethics Committee II of Seth G.S. Medical College and K.E.M. Hospital, Mumbai, India (EC/OA-134/2015). Written informed consent was obtained from each patient before inclusion. Adult patients (>18 years) with unilateral lipid-poor (basal HU > 10) adrenal mass (>1 cm in diameter) who agreed to undergo a CT scan of the abdomen in a protocolized manner were prospectively enrolled from April 2016 to November 2022. Patients were offered standard care as per the diagnosis of the adrenal mass. For the final analysis, only adrenal masses with tissue diagnosis were included.

The final diagnosis of the unilateral adrenal mass was based on primary tumor histopathology and/or adrenal biopsy. Adrenal adenoma was differentiated from ACC (score ≥3) based on the Modified Weiss system [12]. Baseline demographic features and preoperative hormonal profiles were recorded for each patient. An adrenal mass was considered cortisol secreting if overnight dexamethasone-suppressed serum cortisol was ≥1.8 μg/dL, aldosterone secreting as per Endocrine Society guidelines [13], and androgen secreting if serum androgens [dehydroepiandrosterone sulfate/testosterone (T)] were above the upper limit of reference range. Pheochromocytoma was classified as metanephrine secreting (increased plasma free metanephrine with/without elevation of plasma free normetanephrine), normetanephrine secreting (elevated plasma free normetanephrine but normal plasma free metanephrine), or nonsecretory (normal plasma free metanephrines).

### Assays

Plasma free metanephrines were measured using an enzyme immunoassay commercial kit manufactured by Labour Diagnostic (Nord GmbH, Nordhorn, Germany; RRID:AB_2940855). Serum FSH, LH, total T, and cortisol levels were estimated by chemiluminescence immunoassay on the ADVIA Centaur XP platform (Siemens). Plasma direct renin concentration and ACTH levels were measured using solid-phase competitive chemiluminescence immunoassay (LIASON; DiaSorin Inc). Serum dehydroepiandrosterone sulfate was measured by chemiluminescence microparticle immunoassay on the Roche Cobas platform. The intra- and interassay coefficients of variation were <10% for all assays.

### CT Protocol

Imaging of the abdomen was performed on a 64-slice multidetector CT system (Brilliance 64, Philips Healthcare) using a standardized protocol. Patients were placed in the supine position, with arms pulled caudally. The scanning protocol consisted of 4 identical helical scans obtained in an automated, predetermined, and timed sequence. Scanning parameters were 120 kVp, with automatic exposure control (range, 140-220 mA), 0.75 seconds rotation time, a pitch of 0.797, and a 0.625 mm detector configuration with a beam width of 40 mm.

The first phase was obtained at baseline (UP). For patients weighing <60 kg, 60 mL of iodinated contrast (Iohexol: Omnipaque 300, GE Healthcare) was used, while for those ≥60 kg, weight-based dosing of 1 mL/kg was injected into the antecubital vein through a preplaced 18-gauge cannula at a rate of 3 mL/second, followed by a saline flush. Using the bolus tracking technique, the second [early arterial phase (EAP)] phase was obtained when the contrast reached the abdominal aorta at the level of renal artery with a threshold trigger of 180 HU. The third [early venous phase (EVP)] and fourth [delayed venous phase (DVP)] phases were obtained at 45 seconds and 15 minutes, respectively, after the EAP.

All scans were stored on a mass storage device (Seagate, Cupertino, CA, USA) and retrieved by attaching the device to a picture archiving and communication system as a direct storage device. Images were transferred back to the picture archiving and communication system. Images were reconstructed in a standard radiology workstation in transaxial, sagittal, and coronal projections.

### Imaging Analysis

An experienced radiologist, blinded to histopathological diagnosis, reported the CT images on the workstation. Morphological parameters such as size (maximum diameter in axial images) and presence of necrosis, hemorrhage, and calcification were noted. For each phase, attenuation in HU was measured by placing an elliptic region of interest (ROI) of a minimum of 1 cm² at 3 separate sections of the mass, and the mean of these 3 values was calculated. ROI was placed on maximally enhancing areas in the EAP and replicated in identical locations for other (UP, EVP, and DVP) phases. For ROI placement, care was taken to cover the maximum possible area after excluding areas of calcification, necrosis, hemorrhage, and surrounding fat. Additionally, aortic HU (at the level of the renal artery) was measured in EAP.

Relative percentage washout [RPW = (EVP HU − DVP HU) × 100/EVP HU] and absolute percentage washout [absolute percentage washout = (EVP HU − DVP HU) × 100/(EVP HU − UP HU)] were calculated. Percentage arterial enhancement (PAE) was calculated as PAE = (EAP HU − UP HU) × 100/UP HU. Percentage venous enhancement was calculated as percentage venous enhancement = (EVP HU − UP HU) × 100/UP HU.

The adrenal masses were divided into 2 subcohorts: good washout mass (RPW ≥ 40%) and poor washout mass (RPW < 40%). In each subcohort, the radiological characteristics were compared for varied etiologies (adenoma, pheochromocytoma, ACC, and metastatic infiltration/infection) based on the histopathological diagnosis.

### Statistical Analysis

Statistical analysis was performed using SPSS version 26 (IBM). Categorical variables were expressed as absolute number and percentages, and the data was compared using the chi-square and Fisher's exact tests. Continuous variables were expressed as median and interquartile range. Analysis of continuous variables between the 2 groups was carried out using an unpaired *t*-test/Mann–Whitney test, while a 1-way ANOVA test was used to compare more than 2 groups. Receiver operating characteristic (ROC) curves were used to determine the cut-off value(s) for different contrast-enhanced CT parameters that best differentiate varied etiologies of adrenal masses in good washout and poor washout sub-cohorts. A *P*-value <.05 was considered statistically significant.

## Results

**Figure 1.**
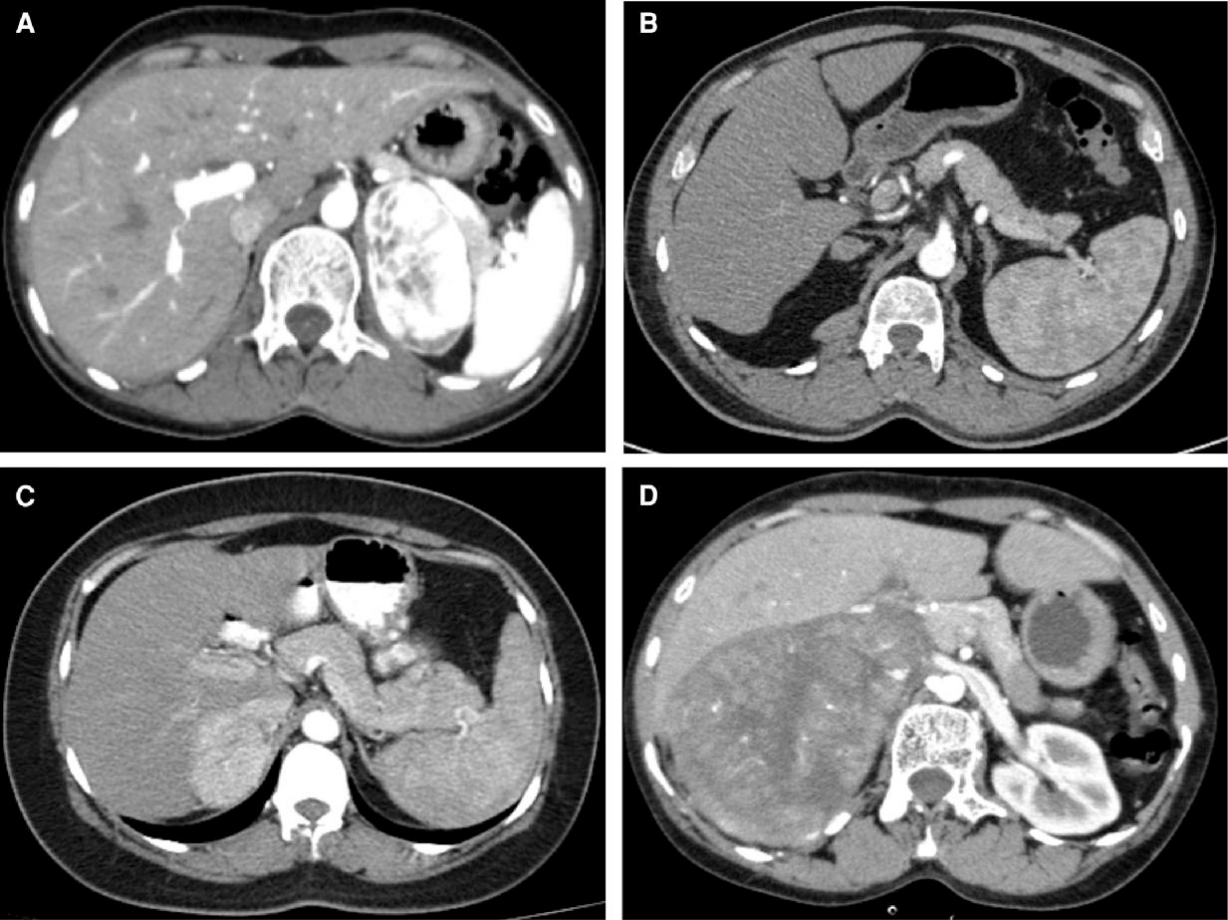
Bolus-tracked arterial phase imaging characteristics. (A) 6.2 cm normetanephrine-secreting left pheochromocytoma with good washout and an attenuation of 284.2 HU; (B) 1.89 cm aldosterone-secreting right adenoma with good washout and an attenuation of 48.6 HU; (C) 5.6 cm normetanephrine-secreting right pheochromocytoma with poor washout and an attenuation of 153.3 HU; and (D) 11.5 cm right adrenocortical carcinoma with poor washout and an attenuation of 62.7 HU.

Abbreviation: HU, Hounsfield unit.

A total of 72 patients (32 males) with a median age of 54 years (range: 18-72 years) were included in the study cohort. The diagnoses based on histopathology were pheochromocytoma (n = 35), ACC (n = 15), lipid-poor adenoma (n = 13), and metastatic infiltration/infection (n = 9). Representative bolus-tracked arterial phase images have been provided in Fig. 1. The detailed clinical and baseline secretory parameters are provided in Table 1. The detailed clinical and baseline secretory parameters are specified in Table 1. Of the 35 pheochromocytomas, 20 (57.1%) were normetanephrine-secreting, 10 (28.6%) were metanephrine-secreting, while the remaining 5 were non-secretory. Based on washout patterns, 15 (42.8%) exhibited good washout, predominantly those secreting normetanephrine (n = 12). In contrast, the poor washout group had almost equal representation of normetanephrine (n = 8) and metanephrine (n = 9) secreting tumors. Patients with ACC had larger tumors (tumor dimension range: 4.8-20 cm); the majority were secretory (n = 13, 86.6%), and all but one had poor washout. Most lipid-poor adenomas had good washout (9/13, 69.2%), and 8 (61.5%) had hormone hypersecretion. The infiltration cohort comprised metastases (n = 5, mostly originating from the lung and one from alveolar soft tissue sarcoma), lymphoma (n = 2), and one each with tuberculosis and myeloma.

**Table 1.** Baseline characteristics of patients included in the study

	Lipid poor adenoma (n = 13)	Pheochromocytoma (n = 35)	Adrenocortical carcinoma (n = 15)	Metastatic infiltration/infection (n = 9)
Age	36 (23-51)	40 (30-52)	36 (22-39)	52 (50-67)
Sex (male/female)	3/10	17/18	5/10	7/2
Biochemical evaluation (%)				
Only cortisol hypersecretion	5/13 (38.5)	0/35 (0)	6/15 (40)	0/9 (0)
Aldosterone hypersecretion	2/13 (15.4)	0/35 (0)	1/15 (6.7)	0/9 (0)
Androgen hypersecretion	1/13 (7.6)	0/35 (0)	1/15 (6.7)	0/9 (0)
Cortisol and androgen hypersecretion	0/13 (0)	0/35 (0)	5/15 (33.3)	0/9 (0)
PFNMN hypersecretion	0/13 (0)	20/35 (57.1)	0/15 (0)	0/9 (0)
PFMN hypersecretion	0/13 (0)	10/35 (28.6)	0/15 (0)	0/9 (0)
Imaging				
Good washout (RPW ≥ 40%)/poor washout (RPW < 40%)	9/4	15/20	1/14	0/9
Largest diameter (cm)	3.4 (2.8-3.7)	5.9 (5.0-7.0)	11.3 (9.0-15.4)	5.1 (3.9-9.9)
Left/right	6/7	16/19	8/7	3/6
Necrosis (%)	4/13 (30.8)	24/35 (68.6)	15/15 (100)	3/9 (33.3)
Hemorrhage (%)	0/13 (0)	2/35 (5.7)	2/15 (13.3)	0/9 (0)
Calcification (%)	0/13 (0)	3/35 (8.6)	4/15 (26.7)	1/9 (11.1)
Enhancement patterns (HU)				
Plain	30.8 (21.3-36.1)	36.6 (34.6-41.9)	42.2 (35.5-43.3)	37.2 (31.8-38.4)
Arterial	69.7 (56.4-81.7)	130.9 (91.9-173.0)	60.4 (55.2-91.6)	46.6 (37.4-71.1)
Venous	82.7 (76.8-93.8)	107.8 (82.2-124.8)	69.7 (62.4-81.9)	63.4 (58.9-87.1)
Delayed	52.7 (38.0-59.1)	66.7 (58.3-75.5)	55.1 (49.9-61.7)	59.9 (50.6-70.9)
Washout patterns (%)				
APW	60.4 (48.2-70.1)	61.2 (50.7-66.5)	53.8 (36.5-66.7)	32.8 (7.0-64.3)
APW > 60%	7/13 (53.8)	18/35 (51.4)	6/15 (40)	3/9 (33.3)
RPW	40.9 (23.9-50.7)	38.9 (26.3-45.5)	25.1 (11.8-29.9)	17.3 (3.2-24.1)
Indices				
PAE (%)	162.9 (97.9-200.5)	237.4 (110.8-392.6)	49.3 (33.9-124.4)	43.4 (5.9-95.2)
PVE (%)	229.0 (144.3-298.4)	193.0 (93.1-256.2)	79.8 (53.3-104.3)	77.8 (59.0-132.2)
Aortic arterial (HU)	254.2 (207.8-309.2)	260.6 (208.0-289.9)	249.6 (216.8-350.9)	256.3 (222.2-321.9)
Arterial HU of mass/aorta	0.28 (0.22-0.32)	0.49 (0.35-0.67)	0.26 (0.20-0.32)	0.18 (0.12-0.25)
Arterial HU > venous HU by ≥5 (%)	1/13 (7.7)	26/35 (74.3)	4/15 (26.7)	1/9 (11.1)
Arterial HU > venous HU by ≥10 (%)	1/13 (7.7)	23/35 (65.7)	3/15 (20)	1/9 (11.1)

Data is expressed as n/N (%) or median (interquartile range).

Abbreviations: APW, absolute percentage washout; HU, Hounsfield unit; PAE, percentage arterial enhancement; PFMN, plasma free metanephrine; PFNMN, plasma free normetanephrine; PVE, percentage venous enhancement; RPW, relative percentage washout.

### CT Characteristics of Good Washout Masses

**Figure 2.**
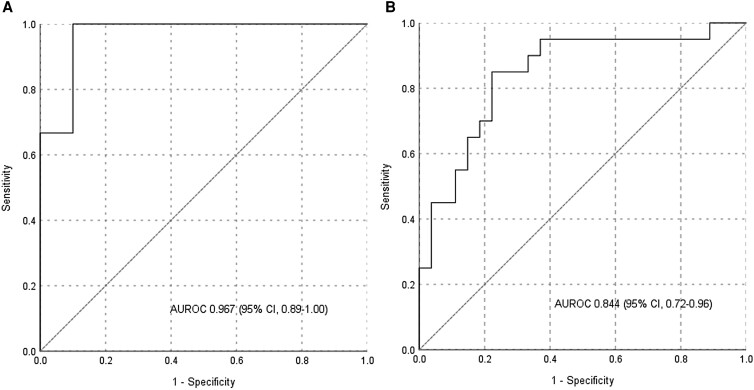
(A) ROC curve for arterial attenuation in good washout pheochromocytomas (relative percentage washout ≥40%) compared with other masses; (B) ROC curve for arterial attenuation in poor washout pheochromocytomas (relative percentage washout <40%) compared with other masses.

Abbreviations: AUROC, area under the receiver operating characteristic curve; CI, confidence interval; ROC, receiver operating characteristic.

The CT characteristics of good washout masses are detailed in Table 2. The lipid-poor good washout masses were mainly either adenomas or pheochromocytomas and one ACC. The ACC was non-secretory with RPW 41%, a largest diameter of 9.88 cm, and a modified Weiss score of 6. Among good washout masses, pheochromocytomas had significantly larger diameters (6.00 vs 3.45 cm, *P* = .001) and higher frequency of necrosis (73.3% vs 30%, *P* = .049). The basal attenuation values were similar across pheochromocytomas and other good washout masses. However, pheochromocytomas exhibited significantly higher post-contrast attenuation across all phases (arterial, venous, and delayed), particularly in the arterial phase (173.2 vs 74.5, *P* < .001). About 86.7% of pheochromocytomas had higher enhancement in the arterial phase relative to the venous phase, with a comparable proportion being 20% for adenomas. The calculated indices, namely percentage arterial enhancement and adrenal mass HU to aorta HU ratio, were also significantly higher in pheochromocytomas. Arterial attenuation provided the highest discriminatory AUC (0.967) among the post-contrast attenuation values (Fig. 2A), with cutoffs of 87.6 HU providing 100% sensitivity and 139.9 HU providing 100% specificity. The novel ratio of arterial attenuation of mass to aorta had an even higher discriminatory value with AUC of 0.987 (Table 3).

**Table 2.** Radiological characteristics of good washout (relative percentage washout ≥ 40%) and poor washout (relative percentage washout < 40%) unilateral lipid-poor adrenal masses

	Good washout			Poor washout		
	Pheochromocytoma (n = 15)	Others (n = 10) [lipid-poor adenoma (n = 9), ACC (n = 1)]	*P*-value	Pheochromocytoma (n = 20)	Others (n = 27) [Lipid poor adenoma (n = 4),ACC (n = 14), Infiltration (n = 9)]	*P* value
Largest diameter (cm)	6.0 (3.8-7.7)	3.4 (2.9-3.8)	.001***^a^***	5.8 (5.3-6.9)	8.6 (3.9-11.9)	.135
Left/right	6/9	6/4	.428	10/10	11/16	.566
Necrosis (%)	11/15 (73.3)	3/10 (30)	.049***^a^***	13/20 (65)	19/27 (70.4)	.758
Hemorrhage (%)	1/15 (6.7)	0/10 (0)	1.000	1/20 (5)	2/27 (7.4)	1.000
Calcification (%)	2/15 (13.3)	1/10 (10)	1.000	1/20 (5)	4/27 (14.8)	.377
Enhancement patterns (HU)						
Plain	34.7 (32.5-37.4)	27.7 (19.5-35.6)	.080	40.9 (35.7-46.5)	37.7 (33.3-42.2)	.102
Arterial	173.2 (123.1-207.6)	74.5 (63.6-81.2)	<.001***^a^***	99.2 (78.1-135.1)	59.2 (49.0-71.4)	<.001***^a^***
Venous	137.2 (107.5-143.9)	89.5 (78.5-96.5)	<.001***^a^***	90.1 (75.3-109.6)	69.7 (60.7-79.1)	.001***^a^***
Delayed	67.6 (54.3-78.1)	47.2 (34.9-53.4)	<.001***^a^***	61.6 (58.6-70.1)	58.2 (50.4-63.2)	.013***^a^***
Washout patterns (%)						
APW	65.0 (58.9-69.3)	68.9 (61.3-73.9)	.397	53.5 (38.8-63.8)	43.7 (28.3-61.0)	.237
APW > 60%	11/15 (73.3)	8/10 (80)	1.000	7/20 (35)	8/27 (29.6)	.758
RPW	45.6 (43.4-48.7)	44.5 (40.9-52.9)	.605	26.9 (22.2-35.2)	22.3 (11.8-25.8)	.009***^a^***
Indices						
PAE (%)	341.3 (264.4-526.0)	162.1 (110.2-211.0)	<.001***^a^***	129.7 (90.4-234.0)	49.3 (28.7-109.6)	<.001***^a^***
PVE (%)	260.8 (228.9-311.6)	215.5 (158.0-358.5)	.367	96.0 (78.5-196.1)	79.8 (60.7-117.2)	.030***^a^***
Aortic arterial (HU)	245.6 (179.8-302.4)	263.3 (218.6-308.4)	.428	269.7 (223.6-289.5)	251.1 (216.8-330.5)	.591
Arterial HU of mass/aorta	0.675 (0.531-0.841)	0.282 (0.232-0.315)	<.001***^a^***	0.418 (0.266-0.498)	0.241 (0.187-0.303)	.001***^a^***
Arterial HU > venous HU by ≥5 (%)	13/15 (86.7)	2/10 (20)	.002***^a^***	13/20 (65)	5/27 (18.5)	.002***^a^***
Arterial HU > venous HU by ≥10 (%)	12/15 (80)	1/10 (10)	.001***^a^***	11/20 (55)	4/27 (14.8)	.005***^a^***

Data is expressed as n/N (%) or median (interquartile range)

Abbreviations: ACC, adrenocortical carcinoma; APW, absolute percentage washout; HU, Hounsfield unit; PAE, percentage arterial enhancement; PFMN, plasma free metanephrine; PFNMN, plasma free normetanephrine; PVE, percentage venous enhancement; RPW, relative percentage washout.

^*a*^ *P* < .05 is considered clinically significant.

**Table 3.** Diagnostic performance of radiological characteristics of good washout (relative percentage washout ≥ 40%) and poor washout (relative percentage washout < 40%) unilateral lipid poor adrenal masses

	Good washout				Poor washout			
	AUC	Value	Sensitivity (%)	Specificity (%)	AUC	Value	Sensitivity (%)	Specificity (%)
Largest diameter (cm)	0.880	3.55	100	70	0.371	—	—	—
Arterial (HU)	0.967	87.6	100	90	0.844	61.7	95	63
		139.9	66.7	100		135.0	75	100
Venous (HU)	0.953	97.7	93	90	0.780	—	—	—
Delayed (HU)	0.900	54.1	86.7	90	0.715	—	—	—
PAE (%)	0.900	218.8	93.3	80	0.800	72.2	90	67
Arterial HU of mass/aorta	0.987	0.327	100	80	0.789	0.308	70	78
		0.441	93.3	100		—	—	—

Abbreviations: AUC, area under curve; HU, Hounsfield unit; PAE, percentage arterial enhancement.

### CT Characteristics of Poor Washout Masses

**Figure 3.**
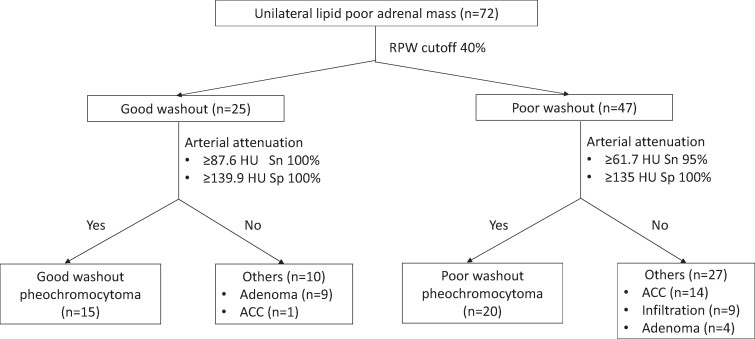
Flow chart of the lipid poor adrenal masses categorized as good or poor washout along with discriminatory cutoffs.

Abbreviations: ACC, adrenocortical carcinoma; HU, Hounsfield unit; RPW, relative percentage washout; Sn, sensitivity; Sp, specificity.

In the poor washout cohort, ACC and metastatic infiltration/infection were similar to each other, except that ACC had a larger diameter (Supplementary data) [14]. The attenuation values post-contrast administration were similar among the adenoma, ACC, and infiltration cohorts; hence, the data was analyzed as poor washout pheochromocytoma vs other poor washout masses (Table 2). Pheochromocytomas had significantly higher arterial (99.2 vs 59.2 HU, *P* < .001), venous, and delayed phase attenuation, as well as higher ratios of percentage arterial/venous enhancement and mass-to-aorta arterial attenuation. Arterial attenuation in pheochromocytomas provided the highest discriminatory AUC (0.844) (Fig. 2B), with a cutoff value of 73.3 HU yielding a sensitivity of 85% and specificity of 78% (Table 3). Within the poor washout group, normetanephrine-secreting/non-secretory pheochromocytomas (n = 11) demonstrated significantly higher arterial enhancement (132.0 vs 90.5 HU, *P* = .020) and mass-to-aorta ratio (0.497 vs 0.241, *P* < .001) compared to metanephrine-secreting pheochromocytomas (n = 9) (Supplementary data) [14]. Figure 3 illustrates the categorization of masses and their respective discriminatory cutoff values across both good and poor washout categories.

## Discussion

In this single-center prospective study of lipid-poor unilateral adrenal masses with bolus-tracked arterial phase imaging and histopathological diagnoses, a sub-cohort of pheochromocytomas exhibited good washout, while a small fraction of adenomas demonstrated poor washout. Good-washout pheochromocytomas could be discriminated from good-washout adenomas based on arterial attenuation cutoffs. Among poor-washout masses, pheochromocytoma had distinctly higher arterial phase attenuation compared to adenoma, ACC, and metastases, albeit with a slightly lower discriminatory power than in good washout masses.

In our cohort of lipid-poor masses, pheochromocytomas were the most common (48.6%), followed by ACC and lipid-poor adenomas, with infiltrative masses being the least common. An epidemiological study of adrenal incidentalomas reported a different composition with adenomas comprising 88.6% of cases reflecting differences in cohort selection [15]. Among the pheochromocytomas, 43% demonstrated good washout (RPW ≥ 40%), most being normetanephrine-secreting. A meta-analysis found good washout characteristics in 35% of pheochromocytomas [6], but did not report secretory parameters. ACCs predominantly showed poor washout, except one non-secretory 9.8 cm mass (41% RPW). Zhang et al noted similar large ACCs (n = 3, RPW ≥ 40%), underscoring the need for additional differentiating parameters [16]. Approximately one-third of lipid-poor adenomas demonstrated poor washout, consistent with previous findings [8]. All the metastatic/infiltrative masses had poor washout, as they did not include renal or hepatic carcinoma metastases (which may show good washout) [17].

Among lipid-poor, good-washout masses, the main differentials remain adenomas and pheochromocytomas, with exceptional ACC. Previous studies have attempted to differentiate adenomas from pheochromocytomas on the basis of washout patterns, with adenomas displaying higher washout [18, 19], though direct comparison between good-washout pheochromocytomas and adenomas has been limited. Our data showed significantly higher arterial phase enhancement (on bolus tracking) in pheochromocytomas, in line with Northcutt et al's report of arterial enhancement >110 HU being suggestive of pheochromocytomas [20]. However, their study was retrospective and predominantly involved lipid-rich adenomas proven by CT follow-up. The progressive enhancement of adenomas in the venous phase (85% in Northcutt's study, 77% in our study), may support the routine inclusion of the early arterial phase in adrenal CT protocols to differentiate from pheochromocytomas. Arterial attenuation value demonstrated a superior AUC of 0.967, with 100% sensitivity at a cutoff of 87.6 HU and 100% specificity at 139.9 HU.

A recent large retrospective study also confirmed significantly higher the arterial attenuation of pheochromocytomas compared to adenomas; however, the PAE was lower [21]. In our study, the PAE was significantly higher in pheochromocytomas probably due to the separation of good and poor washout sub-cohorts which is a more routinely encountered scenario (with availability of delayed phase). Higher venous phase enhancement in pheochromocytomas observed in our study has been previously described [22, 23]. The ratio of arterial attenuation of the mass to the aorta had a better discriminatory value and may have wider applicability as arterial phase acquisition may not be uniform across different centers.

In our study among the poor washout lipid poor masses, pheochromocytomas exhibited significantly higher enhancement on all post-contrast phases, with arterial attenuation providing the best discriminatory value. In the two other studies which attempted distinction between these, the early arterial phase was not evaluated [18, 19]. The overlap and nondiscriminatory venous phase enhancement in both studies contrasts with our findings, probably due to mix of good and poor washout pheochromocytomas and the inclusion of hypervascular metastases from renal cell carcinoma. Pheochromocytomas also demonstrated a higher percentage of arterial and venous enhancement, similar to a prior cohort from our institution, although a distinction between good and poor washout tumors was not attempted at that time [24]. The AUC for arterial attenuation value and the ratio of arterial attenuation of mass compared to aorta was lower than that observed in good washout masses. This could be due to the presence of metanephrine-secreting pheochromocytomas, which have lower arterial attenuation value and a lower proportion of peak enhancement in the arterial phase compared to hypoxia pathway-driven hypervascular normetanephrine-secreting cluster 1 pheochromocytomas [24]. The likely explanation could be the different genetic clusters—most normetanephrine-secreting pheochromocytomas are cluster 1-related whereas metanephrine-secreting are cluster 2-related. Involvement of pseudohypoxia pathway in cluster 1-related pheochromocytomas could potentially lead to rich vascularity and consequent higher arterial attenuation in the same. Although normetanephrine-secreting and non-secretory pheochromocytomas account for a small proportion of adrenal incidentaloma, an arterial attenuation of >151 HU is specific for them and excludes the possibility of metanephrine-secreting pheochromocytoma, besides other adrenal masses. The larger size in ACC or higher frequency of adreniform shape in infiltrative/metastatic masses compared to adenomas has been previously reported [24, 25].

Although hormonal work-up is a must for adrenal mass evaluation, this study provides evidence supporting the use of arterial phase imaging in distinguishing pheochromocytomas from other adrenal masses with baseline attenuation of >10 HU. Both arterial attenuation (AUC: 0.967) and plasma free normetanephrine (AUC: 0.969) had good diagnostic efficacy in distinguishing pheochromocytomas from other adrenal lesions in the current study. Notably, the availability of plasma free and/or 24 hours urinary fractionated metanephrines is limited in several resource-constrained countries whereas their reports are often made available 3-7 days later in centers where the testing is performed. In recent years, the majority of the adrenal incidentalomas are diagnosed via triple-phase (unenhanced, arterial and venous) abdominal CT imaging. In such cases, using the arterial phase imaging information helps in the quick and accurate differentiation of pheochromocytomas from other adrenal masses. We evaluated arterial phase with bolus tracking guidance, which is now the standard technique for abdominal CT imaging. Moreover, the additional imaging data may help to appropriately triage the biochemical test, especially in a resource constrained setting. Further, radiology may provide corroborative evidence in a small fraction of non-secretory adrenal masses. The strengths of our study include its prospective design, a uniform CT protocol, and histopathological confirmation of final diagnoses. This contrasts with most previously published studies that relied on interval CT imaging for adenoma diagnosis.

The limitations include the small number of lipid-poor adenomas, most of which were secretory, potentially confounding enhancement patterns. The proportion of non-secretory pheochromocytomas in this current study (5/35, 14.3%) was higher possibly due to a random bias (small sample size) and use of enzyme immunoassay for the measurement of plasma free metanephrines. Nonetheless, the area under the receiver operating characteristic curve of plasma free metanephrines for diagnosis using enzyme immunoassay in the current study (0.969) is comparable to that reported by Weismann et al (0.993) [26]. Furthermore, the study has a larger representation of pheochromocytomas, which are rare tumors accounting for only 1% to 8% of incidentalomas, limiting its utility in a typical incidentaloma population. However, identifying them is imperative to prevent intraoperative hemodynamic instability, especially as biochemical analysis may be inconclusive in some cases. This study is unique in that it includes all etiologies of lipid-poor adrenal masses, subdivided based on their washout, which is a more commonly encountered situation, and attempts to differentiate them.

## Conclusion

Enhancement on bolus-tracked early arterial phase provides superior discriminatory value in identifying pheochromocytomas from adenomas or ACC/metastases in the good and poor washout subcategories, respectively.

## Data Availability

The data that support the findings of this study are available on request from the corresponding author. The data is not publicly available due to privacy or ethical restrictions.
